# Elastic Carbon Aerogels Reconstructed from Electrospun Nanofibers and Graphene as Three-Dimensional Networked Matrix for Efficient Energy Storage/Conversion

**DOI:** 10.1038/srep31541

**Published:** 2016-08-11

**Authors:** Yunpeng Huang, Feili Lai, Longsheng Zhang, Hengyi Lu, Yue-E Miao, Tianxi Liu

**Affiliations:** 1 State Key Laboratory of Molecular Engineering of Polymers, Department of Macromolecular Science, Fudan University, Shanghai 200433, P. R. China; 2 State Key Laboratory for Modification of Chemical Fibers and Polymer Materials, College of Materials Science and Engineering, Donghua University, Shanghai 201620, P. R. China

## Abstract

Three-dimensional (3D) all-carbon nanofibrous aerogels with good structural stability and elasticity are highly desirable in flexible energy storage/conversion devices. Hence, an efficient surface-induced co-assembly strategy is reported for the novel design and reconstruction of electrospun nanofibers into graphene/carbon nanofiber (CNF) composite aerogels (GCA) with hierarchical structures utilizing graphene flakes as cross-linkers. The as-obtained GCA monoliths possess interconnected macropores and integrated conductive networks, which exhibit high elasticity and great structural robustness. Benefitting from the largely increased surface area and charge-transfer efficiency derived from the multi-form firm interconnections (including pillaring, bridging and jointing) between graphene flakes and CNF ribs, GCA not only reveals prominent capacitive performance as supercapacitor electrode, but also shows excellent hydrogen evolution reaction activity in both acidic and alkaline solutions as a 3D template for decoration of few-layered MoSe_2_ nanosheets, holding great potentials for energy-related applications.

Aerogels, especially carbon-based aerogels (CA), have recently attracted intensive attentions due to their intrinsic advantages like high porosity, light weight, good mechanical stability and excellent electrical conductivity, thus realizing their exciting applications in elastic conductors^1^, water treatment^2^,^3^, catalyst supports^4^, energy storage and conversion^5^,^6^,^7^,^8^, etc. More interestingly, introduction of one-dimensional (1D) nanofibrous building blocks into three-dimensional (3D) aerogels can substantially decrease the density and improve the properties of aerogels in all aspects^9^,^10^. Natural structures like spider webs and bone tissues both indicate that 3D networks composed of 1D building blocks possess excellent structural integrity and low density simultaneously. Inspired by this idea, development of macroscopic nanofibrous CA is becoming a promising strategy to achieve high-performance carbon aerogels with excellent structural robustness and high porosity for widespread applications. For example, Yu *et al*.^11^ prepared ultralight, flexible, and fire-resistant carbon nanofiber (CNF) aerogels from bacterial cellulose pellicles in large scale, which can adsorb a wide range of organic solvents and oils with excellent recyclability and selectivity. Oh and coworkers^12^ reported the first attempt to toughen the intrinsically brittle lignin–resorcinol–formaldehyde aerogel and carbon aerogel using nanofibrous bacterial cellulose, achieving carbon monoliths with low density of 0.026 g cm^−3^, good porous structures and high areal capacitance. Despite these progresses, it still remains great challenges in the efficient assembly and controllable organization of fibrous components into hierarchical and robust CA with multi-functions.

Electrospun nanofibers hold great promise as novel nanofibrous building blocks for the assembly of CA by virtue of their easy processing, good mechanical strength, low density, fine flexibility and composition controllability^13^,^14^,^15^,^16^,^17^. However, the direct lamellar deposition of fibers along single orientation during the electrospinning process only results in stacked fibrous membranes rather than macroscopic aerogels^18^,^19^. Some reports suggest that specially designed fiber collectors can improve the 3D formability to some extent^20^,^21^. Nevertheless, the inherent problem that should be confronted with is to explore advanced techniques for the reconstruction of electrospun fibers into a whole monolith with firm bondings or cross-linkings between every single unit, thus guaranteeing the structural integrity and performance stability under harsh environments. In the valuable work of Ding *et al*.^22^, isotropically-bonded nanofibrous aerogels with hierarchical cellular structures and superelasticity were obtained via a novel freeze-shaping reconstruction of electrospun nanofibers, which involves benzoxazine (BA-a) as the crosslinking agent and SiO_2_ nanofiber as the rigid support. This amazing material with ultra-low density of 0.12 mg cm^−3^ exhibited exceptionally excellent mechanical strength and flexibility, along with multi-functions of thermal insulation, sound absorption, emulsion separation, etc. Greiner *et al*.^23^ introduced another crosslinkable polymer, i.e. poly(methylacrylate-co-methyl methacrylate-co-4-methacryloyloxybenzophenone), into electrospun polyacrylonitrile (PAN) nanofibers, followed by UV crosslinking to obtain stable 3D nanofibrous sponges. A challenge still lies ahead is the failure of chemical crosslinking in the polymeric aerogels after carbonization or graphitization process, which severely degrades the integrity and elasticity of the resulted CA. Therefore, reliable and rigid physical bondings are desperately expected to reconstruct electrospun building blocks into a whole carbon monolith.

Graphene oxide (GO) can be facilely assembled into low-density and highly elastic 3D structures due to its extraordinary flexibility and strength, and subsequently pyrolyzed into carbon aerogels for various energy-related applications^24^,^25^. Furthermore, the abundant oxygen-containing functional groups in GO sheets can serve as cross-linkers to bridge themselves together or interconnect with other functionalized building blocks. Herein, the novel reconstruction of conventional electrospun nanofibers into graphene-carbon nanofiber (CNF) composite aerogels (GCA) were reported via a versatile surface-induced co-assembly method and subsequent carbonization (Fig. 1a), in which two-dimensional (2D) graphene nanosheets act as cross-linkers and the 1D electrospun CNF network serves as the rigid skeleton. The resulted macroscopic GCA with hierarchical combination of 1D and 2D nanostructures manifest low density and excellent structural stability under a certain extent of deformation, which is attributed to the multiform physical interconnections between graphene sheets and CNFs. Attributing to the synergistic effect between the two building blocks, the all-carbon GCA shows a high specific capacitance of 180 F g^−1^ at 1 A g^−1^ as the supercapacitor electrode material. Remarkably, few-layered MoSe_2_ nanosheet decorated GCA (GCAM) also exhibits outstanding catalytic activity towards hydrogen evolution reaction (HER) with onset potentials of −0.09 V and −0.12 V in both acidic and alkaline solutions respectively, demonstrating the great potentials of the all-carbon aerogels as light-weight and flexible frameworks for efficient energy storage and conversion applications.

## Results

### Co-assembly of monolithic GCA

The pre-oxidation of PAN fibers is indispensable during the production of PAN-based carbon fibers, which effectively converts the linear PAN molecular chains into aromatic ladder structures, thus rendering oPAN with non-meltable characteristics to prevent the filament from fusing together during carbonization^26^. Therefore, electrospun oPAN nanofibers, rather than PAN nanofibers, are selected as the precursor to construct all-carbon GCA in our protocol mainly based on two considerations: (1) The pre-oxidation process introduces tertiary amino groups onto oPAN chains, which can improve the hydrophilicity of oPAN nanofibers and help form hydrogen bondings with GO sheets using the lone pair electrons of the nitrogen atom, thus facilitating the strong interactions between these two building blocks. (2) The pre-oxidation process ahead of the aerogel fabrication can effectively avoid potential damage or shrinking of GO-oPAN aerogels during the subsequent harsh treatment.

**Figure 1:**
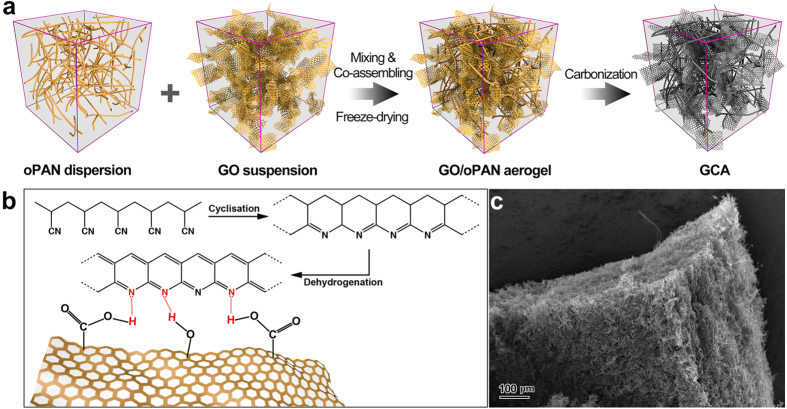
(**a**) Schematic showing the preparation pathway of GCA by co-assembly and carbonization. (**b**) Illustration of the pre-oxidation process of PAN and hydrogen bondings between GO and pre-oxidized PAN. (**c**) SEM image showing the macroscopic GCA.

As shown in Fig. S1, a typical Fourier transform infrared (FTIR) spectrum of PAN exhibits two characteristic peaks at 2239 and 1447 cm^−1^, respectively, which are attributed to the –C≡N stretching vibration and –CH_2_ scissoring vibration according to previous reports^27^,^28^,^29^. After pre-oxidation at 250 °C for 2 h, the intensity of both peaks decreases sharply due to the cyclisation and dehydrogenation during heat treatment (Fig. 1b). It is also noticeable that three new peaks at 1593, 1254 and 795 cm^−1^ emerge in the curve of oPAN, which are ascribed to the C=N group resulted from dehydrogenation, the C–C and C–N stretching vibration, and =C–H out-of-plane wagging vibration, respectively. The appearance of tertiary amino groups in oPAN fibers largely increases the wettability of oPAN membranes with the water contact angle decreased from 117° to 33° (Fig. S2), thus realizing the feasibility of further treatments in aqueous medium. Furthermore, the residual carboxyl groups and hydroxyl groups on the edge of GO sheets can form hydrogen bondings with the amino groups of oPAN fibers (Fig. 1b), playing a key role during the surface-induced co-assembly between oPAN and GO for the construction of robust and compressive all-carbon GCA.

During the synthesis of GCA, the self-standing oPAN nanofibrous membrane with uniform fiber diameter of about 200 nm (Fig. S3a) was first homogenized in water/ethanol mixture to form a well-dispersed brown oPAN dispersion, with the length of short fibers ranging from 40 to 60 μm (Fig. S3b). Then, the oPAN dispersion was mixed with GO suspension (Fig. S4a, with the corresponding atomic force microscopy (AFM) image of monolayer GO shown in Fig. S5) and left to co-assembling under sonication and stirring for enough time. The resulted dark-brown GO/oPAN dispersion was then freeze-dried into GO/oPAN polymeric aerogels in the mould. It’s notable that GO/oPAN aerogels in the centrifuge tube well maintain the cylindrical shape of the mould without noticeable shrinkage observed (Fig. S4b). On the contrary, both freeze-dried oPAN dispersion and GO suspension fail to form stable macroscopic shape, which is probably due to the absence of reliable interactions between the building blocks. X-ray diffraction (XRD) pattern (Fig. S6) of GO/oPAN aerogel indicates the existence of the C (002) peak of GO, while other two peaks at 2θ = 17° and 29.5° are assigned to the crystallite structure of oPAN, which become sharper after pre-oxidation of PAN^30^,^31^. FTIR spectrum of GO/oPAN composite also confirms the presence of C-O (1060 cm^−1^), C-OH (1226 cm^−1^) and C=O (1716 cm^−1^) groups of GO (Fig. S1)^32^.

**Figure 2:**
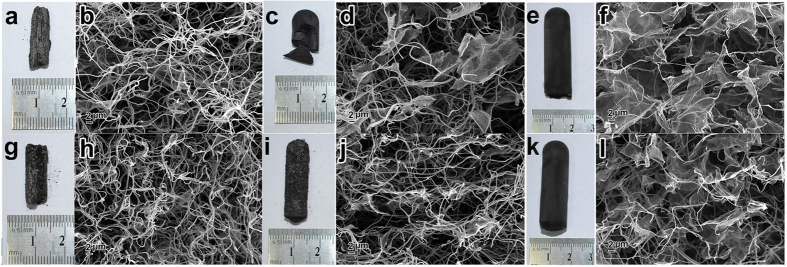
Photographs and SEM images of GCA from different GO:oPAN mass ratios: (**a,b**) GCA12, (**c,d**) GCA22, (**e,f**) GCA42, (**g,h**) GCA14, (**i,j**) GCA24, and (**k,l**) GCA44.

The GO/oPAN polymeric aerogels are finally carbonized into GCA at 800 °C, with oPAN nanofibers converted into carbon nanofibers while GO is simultaneously reduced to form graphene sheets. As shown in Fig. 1c, GCA exhibits a well-defined 3D framework with good formability and structural stability. GO/oPAN aerogels with varying mass ratios of GO to oPAN are obtained, with their macroscopic shapes and corresponding microscopic observations presented in Fig. 2a–l. Obviously, the morphology and structural stability of GCA are highly dependent on the GO:oPAN mass ratio. Under lower GO:oPAN mass ratios, the GCA12, GCA14, and GCA24 aerogels exhibit weak structural integrality in terms of fragility and megascopic porosity (Fig. 2b, h and j). For GCA22, large graphene sheets begin to intertwine between the CNFs (Fig. 2d) with cracks and breakages still exist on the monolith (Fig. 2c), indicating that graphene sheets play an irreplaceable role in the construction of integrated GCA. Further increasing the GO:oPAN mass ratios to 4:2 and 4:4, both GCA42 and GCA44, especially GCA42, display optimized network architectures with interconnected CNFs and expanded graphene sheets uniformly crosslinked with each other (Fig. 2f,l). It is noteworthy that although GCA42 and GCA44 possess the same intact 3D cylindrical structure, the density of GCA42 (4.8 mg cm^−3^) is lower than that of GCA44 (5.9 mg cm^−3^) due to the smaller loading amount of oPAN. Therefore, the GCA mentioned below always represents GCA42 unless specifically stated.

**Figure 3:**
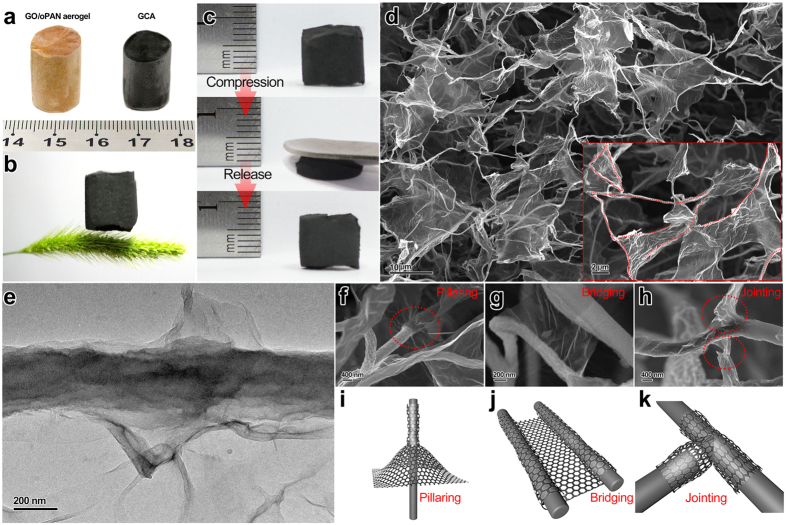
Demonstration on the structural stability and elasticity of GCA with low density, and characterization on the interconnections between graphene and CNF within GCA: (**a**) GCA maintains the good cylindrical shape of GO-oPAN aerogel without noticeable shrinkage, (**b**) a GCA cylinder standing on the soft hair of dog’s tail grass (*Setaria viridis*), and (**c**) GCA aerogel can recover its original shape after a strong compression. (**d**) SEM image of the microscopically porous architecture of GCA, with the inset showing the CNF framework by red dots. (**e**) TEM image of an individual graphene wrapped CNF. (**f–h**) SEM images of multi-form interconnections between graphene and CNFs: (**f**) wrapping, (**g**) pillaring and (**h**) jointing. (**i–k**) Schematic models corresponding to the three kinds of interconnection forms shown in f-h, respectively.

A direct comparison between GO/oPAN aerogels and GCA in Fig. 3a manifests that GCA perfectly retain the integrated 3D structure of GO/oPAN aerogels, suggesting the feasible protocol in this work for synthesis of electrospun nanofiber-based all-carbon aerogels. As delivered in Fig. 3b, the GCA monolith can easily stand on a soft hair of dog’s tail grass (*Setaria viridis*), indicating its low density (4.8 mg cm^−3^). It is also illustrated that GCA can completely recover its original shape without any mechanical fracture after ~70% of compression (Fig. 3c), in sharp contrast to the brittle nature of the traditional colloidal carbon aerogels or foams^3^,^33^. The corresponding compressive stress-strain curves for GCA at the set strains (ε) of 30, 50, and 70% are shown in Fig. S7a. Two distinct regions can be observed during the loading process, *i.e.* the linear elastic region at ε < 60% and the densification region at ε > 60%. Moreover, GCA shows almost complete volume recovery without any plastic deformation after removing the loading force. During the cyclic compressions at the set strain ε = 50% (Fig. S7b), the GCA aerogel can maintain a compressive stress of approximately 4.5 kPa after 30 cycles, indicating the excellent structural robustness of the elastic GCA. In addition, the electrical conductivity of GCA composite is measured to be 9.26 S m^−1^, much higher than that (0.39 S m^−1^) of the pure electrospun carbon nanofiber membrane.

To probe the underlying reasons for the structural robustness and elasticity, close scanning electron microscope (SEM) observations are utilized to study the interconnections between graphene and CNFs. As shown in Fig. 3d, GCA exhibits an interconnected, porous 3D framework with continuous macropores ranging from hundreds of nanometer to tens of micrometer, where short CNFs serving as rigid skeletons are intertwined in random directions while large graphene flakes act as cross-linkers and bridge the CNF ribs to form an integrated network. The inset of Fig. 3d indicates the veins of CNFs ribs by red dots, revealing that CNFs are bridged to form connected pathways which will further enhance the structural stability of GCA. Zooming in image of a single CNF in GCA (Fig. S8b) shows a totally different surface morphology from the smooth surface of neat CNF (Fig. S8a), with the whole CNF tightly wrapped by wrinkled graphene sheets. Transmission electron microscopy (TEM) observation in Fig. 3e further confirms the above assumption by providing a clear-cut profile of CNF enveloped by multiple layers of graphene sheets.

**Figure 4:**
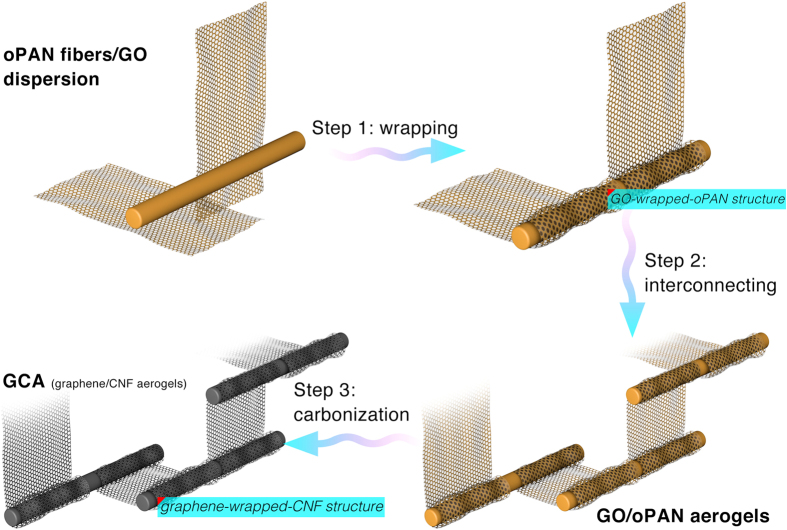
Schematic showing the three-step co-assembly mechanism of GCA.

Based on the above characterizations, we propose a three-step co-assembly mechanism during the preparation of GCA (with a simplified model shown in Fig. 4). In the first step, oPAN fibers are layer-by-layer wrapped by GO sheets through hydrogen bonding to form the tight GO-wrapped-oPAN primary structure. Subsequently, the unwrapped part of GO sheets will interact with other oPAN nanofibers, thus resulting in steady interconnections between individual GO-wrapped-oPAN primary structures. Moreover, the interconnections between GO and oPAN in GO/oPAN aerogels can be completely retained after carbonized to GCA, during which process that GO-wrapped-oPAN primary structures are simultaneously converted into graphene-wrapped-CNF structures. The three-step co-assembly mechanism can also well explain the poor formability and disappearance of graphene sheets in GCA12, GCA14 and GCA24 (Fig. 2b,h,j), in which the second step did not proceed or partly proceeded due to the exhaustion of GO. Detailed observations further proclaim the multi-form interconnections derived from the graphene-wrapped-CNF structures, including pillaring (Fig. 3f), bridging (Fig. 3g), and jointing (Fig. 3h), with the corresponding cartoon models shown in Fig. 3i–k. The multi-form interconnections stemmed from the original GO-oPAN hydrogen bonding play an essential part in the co-assembling of structurally robust and elastic GCA, with the pillaring structure being responsible for supporting and loading bearing while the bridging and jointing structures synergistically protect the building blocks from sliding and splitting under certain tensions.

### Morphology and structure of GCAM composites

**Figure 5:**
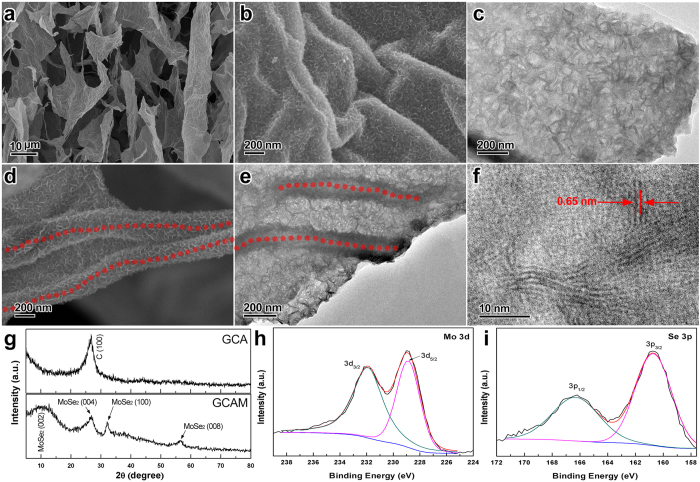
Characterization of GCAM composites. (**a,b,d**) SEM images and (**c,e**) TEM images of GCAM under different magnifications showing the uniform growth of MoSe_2_ on both graphene flakes and the CNF framework (marked by red dots in **d** and **e**). (**f**) HRTEM image of GCAM. (**g**) XRD patterns of GCA and GCAM. (**h,i**) XPS spectra of Mo 3d and Se 3p in GCAM.

Carbon aerogels and foams are potentially applied in energy storage and conversion systems due to their high surface area, intrinsic electrochemical activity and electrical conductivity. Herein, crystalized MoSe_2_ nanosheets are further deposited on GCA through a facile low-temperature solvothermal process combined with post annealing treatment. An overview in Fig. 5a reveals that the GCAM well retains the macoporous structure of GCA without aggregation of MoSe_2_ spheres, indicating that GCA can be excellent substrates to mediate the uniform growth of MoSe_2_. Obviously, MoSe_2_ subunits with curled shape and perpendicular orientation are evenly distributed on both graphene sheets (Fig. 5b,c) and the CNF framework (red dots in Fig. 5d,e). Energy dispersive X-ray spectroscopy (EDS) in Fig. S9 confirms the presence of C, Mo and Se elements in the GCAM composite (Si signal is ascribed to the sample substrate of silicon wafer). In addition, the MoSe_2_ nanosheet is ultrathin as observed from the low contrast with respect to graphene sheet in Fig. 5c,e. A close observation by high-resolution transmission electron microscopy (HRTEM) displays that an individual MoSe_2_ subunit is composed of 4–6 single layers with an interlayer spacing of 0.65 nm (red arrows in Fig. 5f), which matches well with the (002) lattice spacing of MoSe_2_. Undoubtedly, the few-layered subunits will largely increase the exposed active edges of MoSe_2_, thus potentially improving the electrochemical activity of GCAM composite.

Crystal structures of GCAM composite and neat GCA were studied using XRD as shown in Fig. 5g. The broad peak at 2θ = 25.8° in the curve of GCA corresponds to a d-spacing of 3.5 Å, which is consistent with the result for graphene^34^. As for GCAM composite, the peaks at 2θ = 13.7°, 32.2° and 56.4° can be readily indexed to the (002), (100) and (008) diffraction planes of hexagonal 2H-MoSe_2_ phase (JCPDF card, no. 87-2419), respectively. To further determine the oxidation state of MoSe_2_ in GCAM composite, X-ray photoelectron spectroscopy (XPS) analysis is presented in Fig. 5h,i. High-resolution spectrum of Mo 3d reveals two typical peaks at 228.9 and 232.1 eV assigned to Mo 3d_5/2_ and Mo 3d_3/2_ orbitals, confirming the Mo (IV) state. Meanwhile, the binding energies of Se 3p_3/2_ and Se 3p_1/2_ at 160.8 and 166.6 eV indicate the oxidation chemical state of Se^2^,^35^,^36^,^37^. Thermogravimetric analysis (TGA) was conducted under air flow to determine the loading percentage of MoSe_2_ in the GCAM composite. As shown in Fig. S10, pure MoSe_2_ undergoes 9.5% of weight increment between 300 °C and 330 °C, which is in accordance with the previously reported results^38^,^39^. According to the equation of 2MoSe_2_ + 7O_2_ = 2MoO_3_ + 4SeO_2_, the loading percentage of MoSe_2_ in GCAM is calculated to be 28.7% based on the residual weight of MoO_3_.

## Discussion

**Figure 6:**
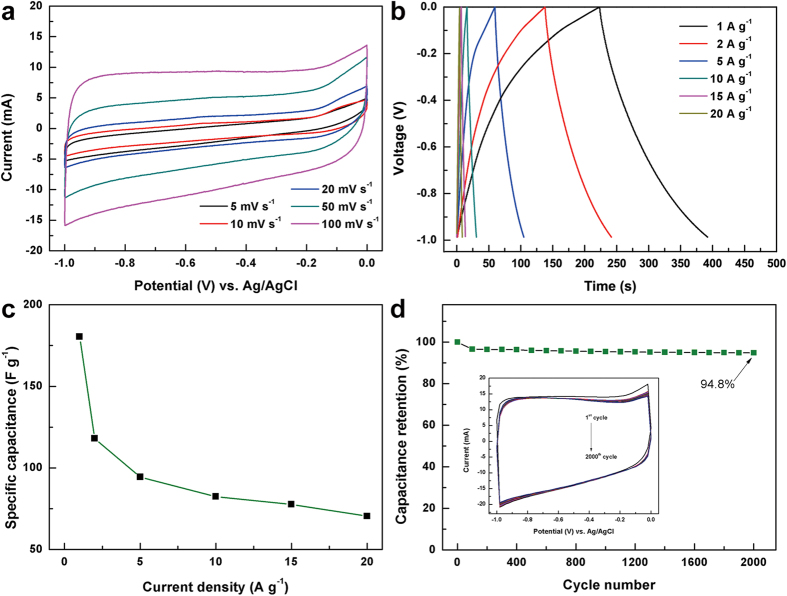
Capacitive tests on GCA electrodes. (**a**) CV curves at different scan rates (5, 10, 20, 50, and 100 mV s^−1^) in 6 M KOH aqueous electrolyte. (**b**) Galvanostatic charge/discharge curves at different current densities (1, 2, 5, 10, 15, and 20 A g^−1^). (**c**) Specific capacitance at different current densities. (**d**) Cycling test of 2000 cycles at 100 mV s^−1^.

The combination of hierarchical 3D structure with numerous macropores, high ion-accessible surface area (57.8 m^2^ g^−1^, as shown in Fig. S11) and interconnected electrically conductive network makes GCA extremely desirable for electrochemical applications. To evaluate the capacitive performance of GCA, Cyclic voltammetry (CV) and galvanostatic charge-discharge measurements were employed. As shown in Fig. 6a, GCA electrode shows nearly rectangular CV shapes under various scan rates, indicating a typical electric double layer capacitor (EDLC) behavior and fast charging/discharging processes. Moreover, it can be discerned that GCA reveals a larger capacitive response over CNFs (Fig. S12a), which is further confirmed by the longer galvanostatic charge-discharge time of GCA than that of CNFs (Fig. 6b and S12b). Especially, the specific capacitance of GCA is about 180 F g^−1^ at 1 A g^−1^, much higher than that (76 F g^−1^ at 1 A g^−1^) of CNFs. In order to investigate the capacitance retention of GCA electrode under high current densities, the variation of specific capacitances at different current densities is summarized in Fig. 6c. Notably, GCA exhibits a high rate capability of 80 F g^−1^ at a current density as high as 20 A g^−1^. Cycling stability test further shows that the GCA electrode keeps high capacitance retention of 94.8% after 2000 cycles (Fig. 6d), which overruns the conventional mesoporous carbon^40^, pure graphene^41^,^42^, and resol-derived carbon/graphene aerogel composites^43^, etc. This is ascribed to the synergistic effect of graphene and CNFs with continuous conductive networks, thus facilitating the fast electron transfer and efficient EDLC energy storage processes.

**Figure 7:**
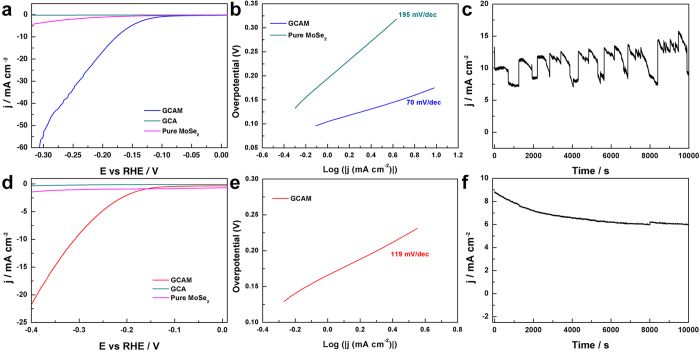
Electrochemical HER activity tests on GCAM modified electrodes in **(a–c)** 0.5 M H_2_SO_4_ and (**d–f**) 1.0 M KOH: (**a,d**) LSV polarization curves for GCA, pure MoSe_2_ spheres, and GCAM composite, (**b,e**) the corresponding Tafel plots, and (**c,f**) time-dependent current density curves at static overpotentials of −0.2 V in 0.5 M H_2_SO_4_ and −0.26 V in 1.0 M KOH, respectively.

The electrocatalytic HER activity of GCAM composite modified GCE in acidic medium was first investigated in 0.5 M H_2_SO_4_ solution using a typical three-electrode setup (Fig. S13). As shown in Fig. 7a, GCAM exhibits a much higher electrochemical activity for HER over neat GCA and pure MoSe_2_ in terms of more positive onset potential (−0.09 V) and higher current density. The corresponding Tafel plots (Fig. 7b) also indicate that the Tafel slop of MoSe_2_ is dramatically decreased from 195 to 70 mV per decade after grown on GCA. To assess the stability of GCAM composite, time-dependent current density curve was collected for 10000 s at a static overpotential of −0.2 V, which demonstrates the excellent long-term stability of GCAM electrode under a constant high current density (Fig. 7c). The superior electrochemical performance suggests that 3D macroporous GCAM composite is an ideal system for HER in acidic solution, which outperforms most of the previously reported non-noble HER catalysts including exfoliated MoS_2_ nanosheets (−0.12 V)^44^, layered MoS_2_ nanosheets obtained by CVD method (−0.15 V)^45^, and S-doped MoSe_2_ nanosheets (−0.1 V)^37^, etc. Additionally, the performance of GCAM composite is also evaluated under alkaline conditions. Figure 7d,e shows the Liner sweep voltammetry (LSV) curves and corresponding Tafel plots for GCAM, GCA and MoSe_2_ in 1.0 M KOH. GCAM exhibits an onset potential of −0.12 V and a Tafel slope of 119 mV per decade, which is comparable to the behaviors of most reported advanced HER catalysts in basic media, *i.e.* bulk MoB^46^, Co-embedded nitrogen-rich carbon nanotubes (CNTs)^47^, and CoP coated carbon cloth^48^. Besides, the time-dependent current density curve (Fig. 7f) at a static overpotential of −0.26 V shows that the GCAM modified electrode retains 70% of its initial current density after 10000 s, indicating the good stability of GCAM composite as a HER catalyst in alkaline solutions. It is noteworthy that pure MoSe_2_ modified GCE exhibits no noticeable HER activity in KOH solution, which is ascribed to the intrinsic poor electrical conductivity and severely decreased active sites of agglomerated MoSe_2_ spheres (Fig. S14). Electrochemical impedance spectroscopy (EIS) results (Fig. S15) further show that GCAM has a much lower Faradaic impedance than pure MoSe_2_ spheres in both acidic and alkaline solution, indicating that the electron transfer rate of MoSe_2_ in HER is greatly enhanced after growing on the GCA framework. All these electrochemical evaluations highlight the successful combination of electro-catalytic MoSe_2_ and the all-carbon networked GCA, which maximizes the exposed active sites and offers electron transfer pathways to synergistically enhance the HER catalytic activity in both acidic and alkaline medium.

In summary, for the first time, a surface-induced co-assembly strategy has been developed for the facile preparation of elastic, macroporous and structural robust GCA through the combination of 1D electrospun nanofibers and 2D graphene sheets. The all-carbon aerogels with monolithic 3D frameworks are constructed by the cell walls of graphene sheets and CNF ribs, which overcomes the intrinsic defect of conventionally deposited 2D electrospun nanofiber membranes. The proposed multi-form interconnection mechanism further indicates that firmly constructed 3D framework can be achieved by a facile co-assembly strategy by virtue of the intimate crosslinking between graphene and CNFs, endowing the co-assembled GCA with prominent capacitive performance as supercapacitor electrode. More importantly, a rationally designed hierarchical composite of few-layered MoSe_2_ doped GCA even manifests high-performance HER catalytic activity in both acidic and alkaline mediums, which further broadens the potential applications of the novel aerogel in various energy-related fields. It is also envisioned that this work will open up the extensive reconstitution of traditional electrospun nanofibers from a wide range of polymers.

## Methods

### Materials

Polyacrylonitrile (PAN, Mw = 150,000 g mol^−1^) was purchased from Sigma–Aldrich. Natural graphite powder (325 meshes) was obtained from Alfa-Aesar. Selenium powder (Se, 99.99%), Na_2_MoO_4_ (99.99%), hydrazine hydrate (N_2_H_4_·H_2_O, 50 wt% in water), *N,N*-dimethylformamide (DMF), ethanol, 98% H_2_SO_4_, 30% H_2_O_2_, KMnO_4_, NaNO_3_, and 37% HCl were provided by Sinopharm Chemical Reagent Co. Ltd. All aqueous solutions were prepared with doubly distilled (DI) water.

### Fabrication of GCA with controllable parameters

PAN nanofibrous membranes were first produced through a facile single-nozzle electrospinning technique using a commercial electrospinning system (UCALERY Beijing Co., LTD, China). Typically, the precursor solution containing 0.1 g mL^−1^ PAN was first prepared by dissolving a specific amount of PAN powder into DMF under magnetic stirring at 80 °C for 5 h. The freshly obtained homogeneous polymer solution was then loaded into a 10 mL plastic syringe and injected with a feeding rate of 0.25 mm min^−1^ through a No. 21 stainless steel needle connected to a high-voltage DC power supply. A rotating aluminum drum was set as the collector with a distance of 15 cm to the needle tip. When a fixed voltage of 20 kV was applied to the system, PAN nanofibers were generated and deposited on the aluminum drum. After 12 h vacuum drying to remove any residual solvents, PAN membranes were then pre-oxidized in air atmosphere under 250 °C for 2 h with a heating rate of 1 °C min^−1^. The pre-oxidized PAN (oPAN) nanofibrous membranes were cut into 2 × 2 cm^2^ pieces and homogenized in the mixed solvent of water/ethanol at 13,000 r.p.m. for 30 min using an IKA T25 homogenizer (Fig. S16). The obtained dark brown oPAN nanofiber dispersion was dehydrated by suction filtration and overnight dried at 70 °C.

Graphite oxide was synthesized by Hummers method^49^ and exfoliated to give a brown dispersion of graphene oxide (GO) under ultrasonication. Then, a certain amount of oPAN short fibers was re-dispersed in DI water under 1 h of sonication and 5 h of magnetic stirring. To fabricate GO/oPAN composites (as depicted in Fig. 1a), equal volumes of GO suspension and oPAN dispersion at the concentration of 1 to 4 mg mL^−1^ were mixed through repetitive magnetic stirring and sonication to assure the sufficient contact and interactions between GO sheets and oPAN nanofibers. The homogeneous GO/oPAN composite dispersion was then transferred into centrifuge tubes (5 mL), frozen in liquid nitrogen (LN) and freeze-dried (FD-1D-50, Beijing Boyikang Lab Instrument Co., Ltd.) for 48 h to obtain GO/oPAN aerogels. Finally, the GO/oPAN aerogels were treated in 800 °C tube furnace for 1 h to yield the macroscopic all-carbon GCA. By adjusting the GO:oPAN mass ratios (1:2, 1:4, 2:2, 2:4, 4:2, and 4:4) in GO/oPAN aerogels, GCA with controllable compositions were produced and denoted as GCA12, GCA14, GCA22, GCA24, GCA42, and GCA44, respectively. In addition, GO suspension and oPAN dispersion were respectively frozen and freeze-dried for comparison.

### Synthesis of GCAM composites

MoSe_2_ nanosheets were grown in GCA via a simple solvothermal method according to our previous work^38^. Briefly, 4 mg mL^−1^ Se solution was first prepared in a flask by adding a certain amount of Se powder into N_2_H_4_·H_2_O. The colorless solution soon turned dark brown after 1 h of magnetic stirring at 80 °C oil bath. In a separate flask, 5 mg of GCA was immersed in 10 mL of DMF solution containing stoichiometric amount of Na_2_MoO_4_. Then, 10 mL of Se solution was dropwise added into the above solution with a final Mo:Se molar ratio of 1:2. Afterwards, the mixture was transferred into a 40 mL Teflon-lined autoclave and heated at 180 °C for 12 h to grow MoSe_2 _on GCA. Thus, GCAM composite monolith was obtained after rinsing with DI water and freeze-drying. Subsequent annealing was performed in N_2_ at 450 °C for 2 h with a ramp rate of 5 °C min^−1^ to yield crystalized GCAM composite. Pure MoSe_2_ was also produced via the above method without the addition of GCA.

### Characterization

Morphology of the samples was investigated using field emission scanning electron microscope (FESEM, Zeiss) at an acceleration voltage of 5 kV. Transmission electron microscopy (TEM) was performed under an acceleration voltage of 200 kV with a Tecnai G2 20 TWIN TEM. Thermogravimetric analysis (Pyris 1 TGA) was performed under air flow from 100 to 700 °C at a heating rate of 20 °C min^−1^. Fourier transform infrared spectra (FT-IR) were measured on a Nicolet 6700 FT-IR spectrometer. X-ray diffraction (XRD) experiments were conducted from 2θ = 10° to 80° on an X’Pert Pro X-ray diffractometer with CuK_α_ radiation (λ = 0.1542 nm) under a voltage of 40 kV and a current of 40 mA. X-ray photoelectron spectroscopy (XPS) analyses were made with a RBD upgraded PHI-5000C ESCA system (Perkin Elmer) with K (1,486.6 eV) as X-ray source. All XPS spectra were corrected using C1s line at 284.6 eV. Curve fitting and background subtraction were accomplished using XPS PEAK41 software. Atomic force microscopy (AFM) images were taken under tapping mode with a Scanning Probe Microscope (SPM) Nanoscope IV from Digital Instruments. The water contact angles were measured using an automatic video contact-angle testing apparatus (Model OCA 40, Dataphysics). The specific surface area of GCA was characterized with a belsorp-max surface area detecting instrument (Tristar3000) by N_2_ physisorption at 77 K. The compressive tests were conducted using a universal testing machine (SANS CMT-4104, Shenzhen, China) equipped with two flat-surface compression stages and a 80 N load cell at a loading rate of 5 mm min^−1^. The electrical conductivity of the samples was collected using a Keithley 4200 semi-conductor system at room temperature.

### Electrochemical measurements

All electrochemical measurements were conducted with a CHI 660D electrochemical workstation (Chenhua instrument Co., Shanghai, China) at room temperature. The capacitive performance of GCA was evaluated in a 6 M KOH aqueous electrolyte with a standard three-electrode setup where a platinum wire served as the counter electrode and a Ag/AgCl electrode as the reference electrode. The working electrodes were simply prepared by pressing a piece of GCA (0.3–0.5 mg) between two nickel foams. Cyclic voltammetry (CV) curves and galvanostatic charge-discharge curves were collected in a voltage ranging from −1 to 0 V. The specific capacitances of all electrodes were calculated from the discharge process according to the following equation:
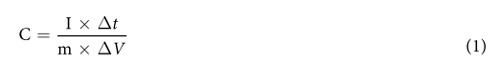
where *I* (A), *m* (g), *∆V* (V), and *∆t* (s) represent the discharge current, the mass of active materials, potential window during the discharge process, and discharge time, respectively.

The electrochemical HER tests of GCAM were performed in an electrolyte solution of 0.5 M H_2_SO_4_ and 1.0 M KOH, respectively. A graphite rod and a saturated calomel electrode (SCE) were used as the counter electrode and the reference electrode, respectively. The working electrode was prepared by pasting a tablettized GCAM (3 mm in diameter) onto the polished glassy carbon electrode (GCE) using 5 μL of Nafion solution (5 wt % in ethanol). Pure MoSe_2_ modified GCE was prepared via dropping 10 μL of MoSe_2_ slurry (1 mg mL^−1^ in DI water) onto GCE and left to dry. Liner sweep voltammetry (LSV) measurements were conducted with a scan rate of 5 mV s^−1^. In all measurements, the SCE reference electrode was calibrated with respect to the reversible hydrogen electrode (RHE) according to E (RHE) = E (SCE) + 0.281 V (in 0.5 M H_2_SO_4_), and E (RHE) = E (SCE) + 1.068 V (in 1.0 M KOH). The onset potential was determined based on the beginning of the linear region in the Tafel plot without iR compensation applied for all the electrochemical measurements. Electrochemical impedance spectroscopy (EIS) was recorded in the frequency ranging from 0.01 Hz to 100 kHz with an amplitude of 5 mV at a cathodic bias that drives rapid hydrogen evolution according to the literature^50^. In addition, the initial electric potential was set as −0.3 V vs. RHE in our work for direct comparison.

## Additional Information

**How to cite this article**: Huang, Y. *et al*. Elastic Carbon Aerogels Reconstructed from Electrospun Nanofibers and Graphene as Three-Dimensional Networked Matrix for Efficient Energy Storage/Conversion. *Sci. Rep.* **6**, 31541; doi: 10.1038/srep31541 (2016).

## Supplementary Material

Supplementary Information

## References

[b1] Ge J. . Stretchable conductors based on silver nanowires: improved performance through a binary network design. Angew. Chem. Int. Ed. 125, 1698–1703 (2013). 10.1002/anie.201209596 23355499

[b2] Bi H. C. . Carbon fiber aerogel made from raw cotton: a novel, efficient and recyclable sorbent for oils and organic solvents. Adv. Mater. 25, 5916–5921 (2013). 24038404 10.1002/adma.201302435

[b3] Gao W. . Ni-doped graphene/carbon cryogels and their applications as versatile sorbents for water purification. ACS Appl. Mater. Interfaces 5, 7584–7591 (2013). 23855959 10.1021/am401887g

[b4] Wu Z. S. . 3D nitrogen-doped graphene aerogel-supported Fe_3_O_4_ nanoparticles as efficient electrocatalysts for the oxygen reduction reaction. J. Am. Chem. Soc. 134, 9082–9085 (2012). 22624986 10.1021/ja3030565

[b5] Chen S. L. . Elastic carbon foam via direct carbonization of polymer foam for flexible electrodes and organic chemical absorption. Energy Environ. Sci. 6, 2435–2439 (2013).

[b6] Kang E. . Fe_3_O_4_ nanoparticles confined in mesocellular carbon foam for high performance anode materials for lithium-ion batteries. Adv. Funct. Mater. 21, 2430–2438 (2011).

[b7] Yin L. W. . Spinel ZnMn_2_O_4_ nanocrystal-anchored 3D hierarchical carbon aerogel hybrids as anode materials for lithium ion batteries. Adv. Funct. Mater. 24, 4176–4185 (2014).

[b8] Zhang Y. F., Fan W., Huang Y. P., Zhang C. & Liu T. X. Graphene/carbon aerogels derived from graphene crosslinked polyimide as electrode materials for supercapacitors. RSC Adv . 5, 1301–1308 (2015).

[b9] Gao H. L. . Macroscopic free-standing hierarchical 3D architectures assembled from silver nanowires by ice templating. Angew. Chem., Int. Ed. 53, 4561–4566 (2014). 10.1002/anie.201400457 24683064

[b10] Yan S. H., Zhen X. & Chao G. Multifunctional, ultra-flyweight, synergistically assembled carbon aerogels. Adv. Mater. 25, 2554–2560 (2013). 23418099 10.1002/adma.201204576

[b11] Wu Z. Y., Li C., Liang H. W., Chen J. F. & Yu S. H. Ultralight, flexible, and fire-resistant carbon nanofiber aerogels from bacterial cellulose. Angew. Chem. Int. Ed. 52, 2925–2929 (2013). 10.1002/anie.201209676 23401382

[b12] Xu X. Z. . Flexible, highly graphitized carbon aerogels based on bacterial cellulose/lignin: catalyst-free synthesis and its application in energy storage devices. Adv. Funct. Mater. 25, 3193–3202 (2015).

[b13] Inagaki M., Yang Y. & Kang F. Carbon nanofibers prepared via electrospinning. Adv. Mater. 24, 2547–2566 (2012). 22511357 10.1002/adma.201104940

[b14] Greiner A. & Wendorff J. H. Electrospinning: a fascinating method for the preparation of ultrathin fibers. Angew. Chem. Int. Ed. 46, 5670–5703 (2007). 10.1002/anie.200604646 17585397

[b15] Huang Y. P. . Synthesis of few-layered MoS_2_ nanosheet-coated electrospun SnO_2_ nanotube heterostructures for enhanced hydrogen evolution reaction. Nanoscale 6, 10673–10679 (2014). 25089760 10.1039/c4nr02014f

[b16] Xiong Z. Y., Kong X. Y., Guo Z. X. & Jian Y. Poly(ethylene terephthalate)/carbon black composite fibers prepared by electrospinning. Chin. J. Polym. Sci . 33, 1234–1244 (2015).

[b17] Abbasi A., Nasef M. M., Takeshi M. & Faridi-majidi R. Electrospinning of nylon-6,6 solutions into nanofibers: rheology and morphology relationships. Chin. J. Polym. Sci . 32, 793–804 (2014).

[b18] Tai M. H., Tan B. Y. L., Juay J., Sun D. D. & Leckie J. O. A self-assembled superhydrophobic electrospun carbon-silica nanofiber sponge for selective removal and recovery of oils and organic solvents. Chem. - Eur. J. 21, 5395–5402 (2015). 25597480 10.1002/chem.201405670

[b19] Ahirwal D., Hebraud A., Kadar R., Wilhelm M. & Schlatter G. From self-assembly of electrospun nanofibers to 3D cm thick hierarchical foams. Soft Mater 9, 3164–3172 (2013).

[b20] Sun B. . Self-assembly of a three-dimensional fibrous polymer sponge by electrospinning. Nanoscale 4, 2134–2137 (2012). 22344309 10.1039/c2nr11782g

[b21] Teo W., Inai R. & Ramakrishna S. Technological advances in electrospinning of nanofibers. Sci. Technol. Adv. Mater. 12, 13002 (2011). 10.1088/1468-6996/12/1/11660944 PMC5090397 27877375

[b22] Si Y., Yu J. Y., Tang X. M., Ge J. L. & Ding B. Ultralight nanofibre-assembled cellular aerogels with superelasticity and multifunctionality. Nat. Commun. 5, 5802 (2014). 25512095 10.1038/ncomms6802

[b23] Duan G. G. . Ultralight, soft polymer sponges by self-assembly of short electrospun fibers in colloidal dispersions. Adv. Funct. Mater. 25, 2850–2856 (2015).

[b24] Liu R. L. . An interface-induced co-assembly approach towards ordered mesoporous carbon/graphene aerogel for high-performance supercapacitors. Adv. Funct. Mater. 25, 526–533 (2015).

[b25] Wang X. . Scalable template synthesis of resorcinol-formaldehyde/graphene oxide composite aerogels with tunable densities and mechanical properties. Angew. Chem., Int. Ed. 54, 2397–2401 (2015). 10.1002/anie.201410668 25583599

[b26] Pierson H. O. Handbook of carbon, graphite, diamond, and fullerenes: properties, processing, and applications (Noyes Publications, 1993).

[b27] Mittal J., Bahl O. P., Mathur R. B. & Sandle N. K. IR studies of PAN fibres thermally stabilized at elevated temperatures. Carbon 32, 1133–1136 (1994).

[b28] Manocha L. M. & Bahl O. P. Role of oxygen during thermal stabilisation of PAN fibres. Fibre Sci. Technol. 13, 199–212 (1980).

[b29] Clarke A. J. & Bailey J. E. Oxidation of acrylic fibres for carbon fibre formation. Nature 243, 146–150 (1973).

[b30] Gupta A. K. & Maiti A. K. Effect of heat treatment on the structure and mechanical properties of polyacrylonitrile fibers. J. Appl. Polym. Sci. 27, 2409–2416 (1982).

[b31] Ji M. X., Wang C. G., Bai Y. J., Yu M. J. & Wang Y. X. Structural evolution of polyacrylonitrile precursor fibers during preoxidation and carbonization. Polym. Bull. 59, 527–536 (2007).

[b32] Mei X. G. & Ouyang J. Y. Ultrasonication-assisted ultrafast reduction of graphene oxide by zinc powder at room temperature. Carbon 49, 5389–5397 (2011).

[b33] Chen H. B., Wang Y. Z. & Schiraldi D. A. Preparation and flammability of poly(vinyl alcohol) composite aerogels. ACS Appl. Mater. Interfaces 6, 6790–6796 (2014). 24731187 10.1021/am500583x

[b34] Liu M. K. . Hierarchical composites of polyaniline-graphene nanoribbons-carbon nanotubes as electrode materials in all-solid-state supercapacitors. Nanoscale 5, 7312–7320 (2013). 23821299 10.1039/c3nr01442h

[b35] Tang H., Dou K. P., Kaun C. C., Kuang Q. & Yang S. H. MoSe_2_ nanosheets and their graphene hybrids: synthesis, characterization and hydrogen evolution reaction studies. J. Mater. Chem. A 2, 360–364 (2014).

[b36] Zhou X. L. . Fast colloidal synthesis of scalable Mo-rich hierarchical ultrathin MoSe_(2-x)_ nanosheets for high-performance hydrogen evolution. Nanoscale 6, 11046–11051 (2014). 25144136 10.1039/c4nr02716g

[b37] Xu C. . Ultrathin S-doped MoSe_2_ nanosheets for efficient hydrogen evolution. J. Mater. Chem. A 2, 5597–5601 (2014).

[b38] Huang Y. P. . Perpendicularly oriented few-layer MoSe_2_ on SnO_2_ nanotubes for efficient hydrogen evolution reaction. J. Mater. Chem. A 3, 16263–16271 (2015).

[b39] Liu Y., Zhu M. Q. & Chen D. Sheet-like MoSe_2_/C composites with enhanced Li-ion storage properties. J. Mater. Chem. A 3, 11857–11862 (2015).

[b40] Lu H. L. . Electrochemical capacitive behaviors of ordered mesoporous carbons with controllable pore sizes. J. Power Sources 209, 243–250 (2012).

[b41] Xu Y. X., Sheng K. X., Li C. & Shi G. Q. Self-assembled graphene hydrogel via a one-step hydrothermal process. ACS Nano 4, 4324–4330 (2010). 20590149 10.1021/nn101187z

[b42] Stoller M. D., Park S. J., Zhu Y. W., An J. & Ruoff R. S. Graphene-based ultracapacitors. Nano Lett. 8, 3498–3502 (2008). 18788793 10.1021/nl802558y

[b43] Qian Y. Q., Ismail I. M. & Stein A. Ultralight, high-surface-area, multifunctional graphene-based aerogels from self-assembly of graphene oxide and resol. Carbon 68, 221–231 (2014).

[b44] Ji S. S. . Exfoliated MoS_2_ nanosheets as efficient catalysts for electrochemical hydrogen evolution. Electrochim. Acta 109, 269–275 (2013).

[b45] Lukowski M. A. . Enhanced hydrogen evolution catalysis from chemically exfoliated metallic MoS_2_ nanosheets. J. Am. Chem. Soc. 135, 10274–10277 (2013). 23790049 10.1021/ja404523s

[b46] Vrubel H. & Hu X. L. Molybdenum boride and carbide catalyze hydrogen evolution in both acidic and basic solutions. Angew. Chem. Int. Ed. 124, 12875–12878 (2012). 10.1002/anie.201207111 23143996

[b47] Zou X. X. . Cobalt-embedded nitrogen-rich carbon nanotubes efficiently catalyze hydrogen evolution reaction at all pH values. Angew. Chem. Int. Ed. 126, 4461–4465 (2014). 10.1002/anie.201311111 24652809

[b48] Tian J. Q., Liu Q., Asiri A. M. & Sun X. P. Self-supported nanoporous cobalt phosphide nanowire arrays: an efficient 3D hydrogen-evolving cathode over the wide range of pH 0–14. J. Am. Chem. Soc. 136, 7587–7590 (2014). 24830333 10.1021/ja503372r

[b49] Hummers W. S. & Offeman R. E. Preparation of graphitic oxide. J. Am. Chem. Soc. 80, 1339 (1958).

[b50] Faber M. S. . High-performance electrocatalysis using metallic cobalt pyrite (CoS_2_) micro- and nanostructures. J. Am. Chem. Soc. 136, 10053–10061 (2014). 24901378 10.1021/ja504099w

