# Supplementary material for: Elastic Carbon Aerogels Reconstructed from Electrospun Nanofibers and Graphene as Three-Dimensional Networked Matrix for Efficient Energy Storage/Conversion
Source: Sci Rep. 2016 Aug 11;6:31541. doi: 10.1038/srep31541 (PMC4980659; doi:10.1038/srep31541)
Supplement: Supplementary Information [file srep31541-s1.doc]

Supporting Information

Elastic Carbon Aerogels Reconstructed from Electrospun Nanofibers and Graphene as Three-Dimensional Networked Matrix for Efficient Energy Storage/Conversion

Yunpeng Huang, Feili Lai, Longsheng Zhang, Hengyi Lu, Yue-E Miao*, Tianxi Liu*

Y. P. Huang, F. L. Lai, L. S. Zhang, H. Y. Lu, Prof. T. X. Liu

State Key Laboratory of Molecular Engineering of Polymers, Department of Macromolecular Science, Fudan University, Shanghai 200433, P. R. China.

Dr. Y. E. Miao, Prof. T. X. Liu
State Key Laboratory for Modification of Chemical Fibers and Polymer Materials, College of Materials Science and Engineering, Donghua University, Shanghai 201620, P. R. China.


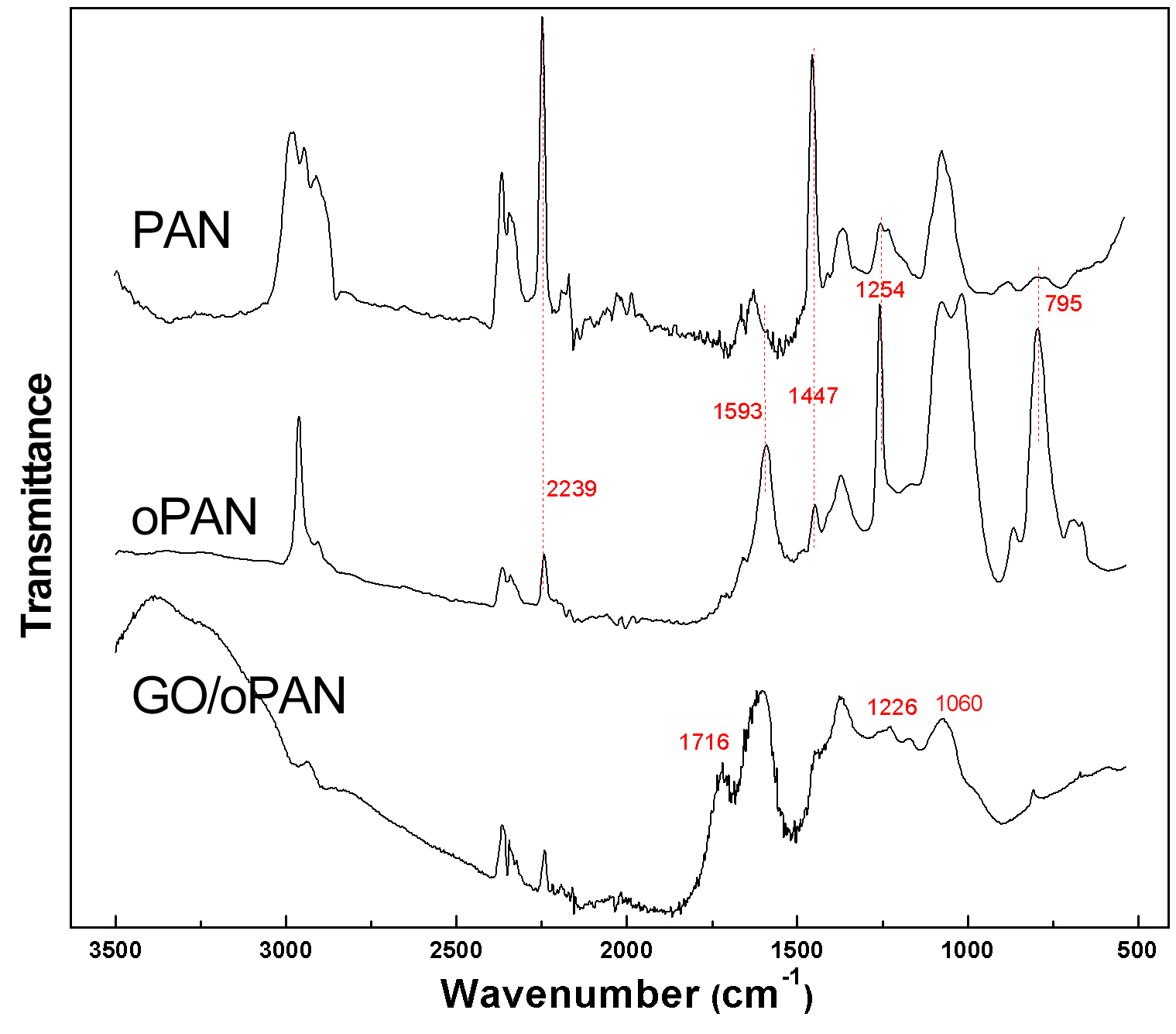


**Figure S1.** FTIR spectra of PAN, oPAN and GO/oPAN composites.


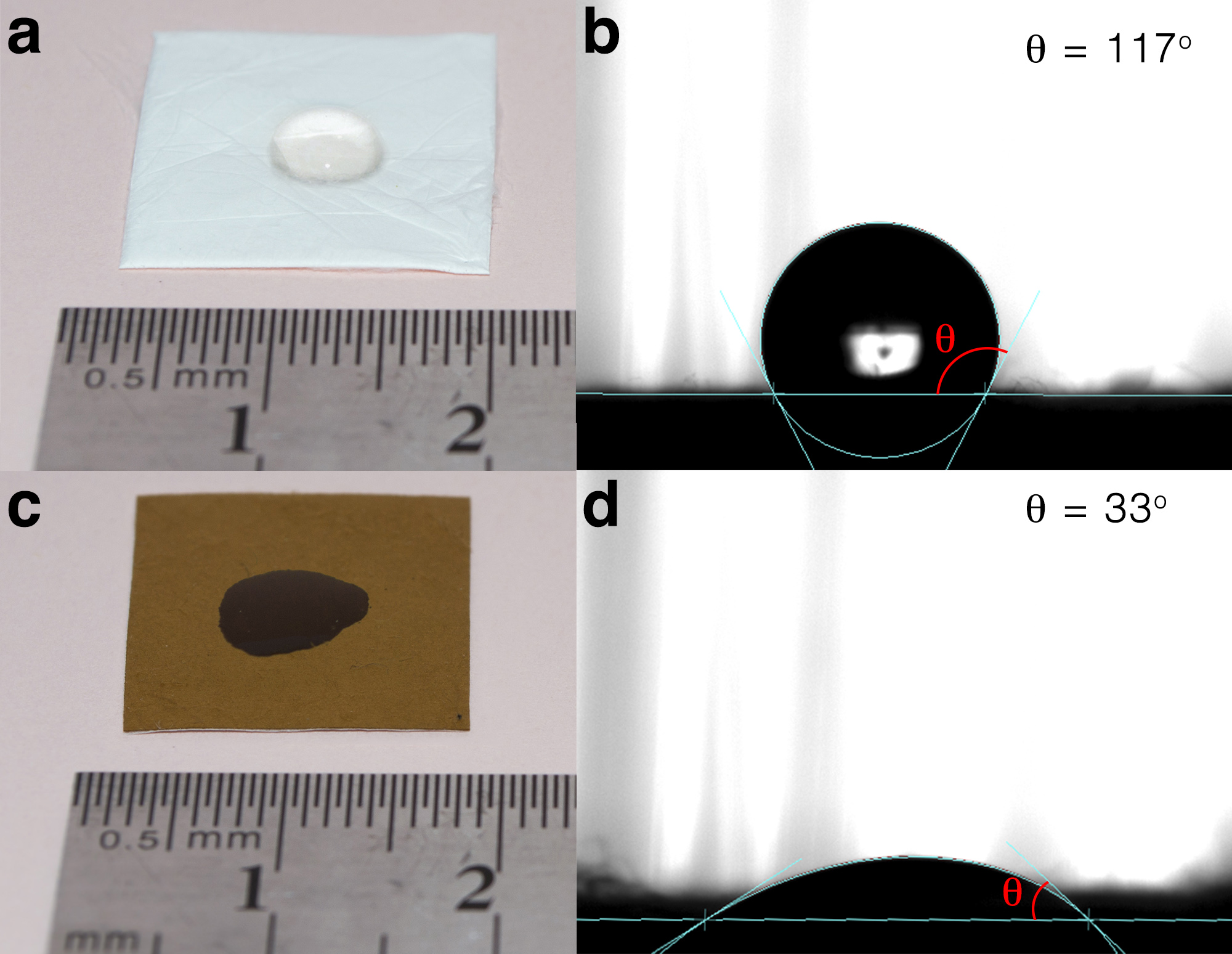


**Figure S2.** Photographs showing the wettability of PAN (a) and oPAN (c) membranes. (b, d) are the corresponding contact angle measurements, showing the wettability of PAN membranes is greatly improved after pre-oxidation.


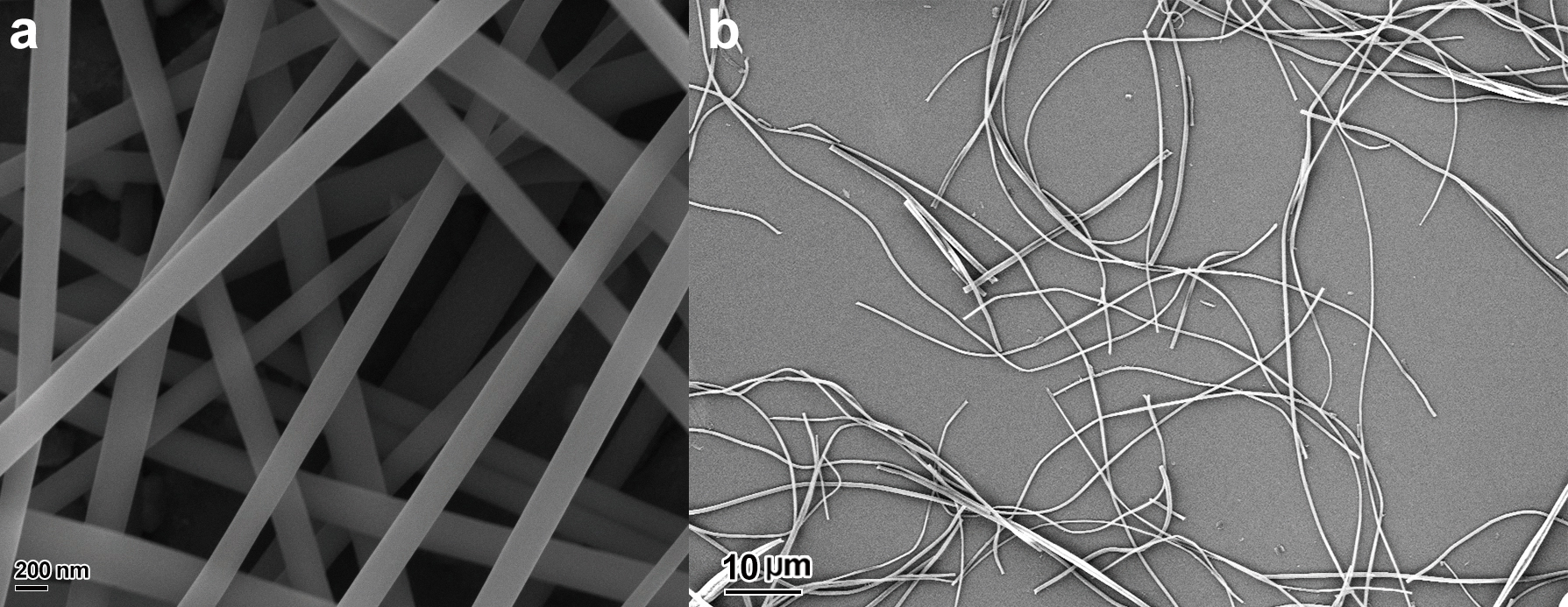


**Figure S3.** SEM images of oPAN nanofibers before (a) and after (b) homogenization treatment.


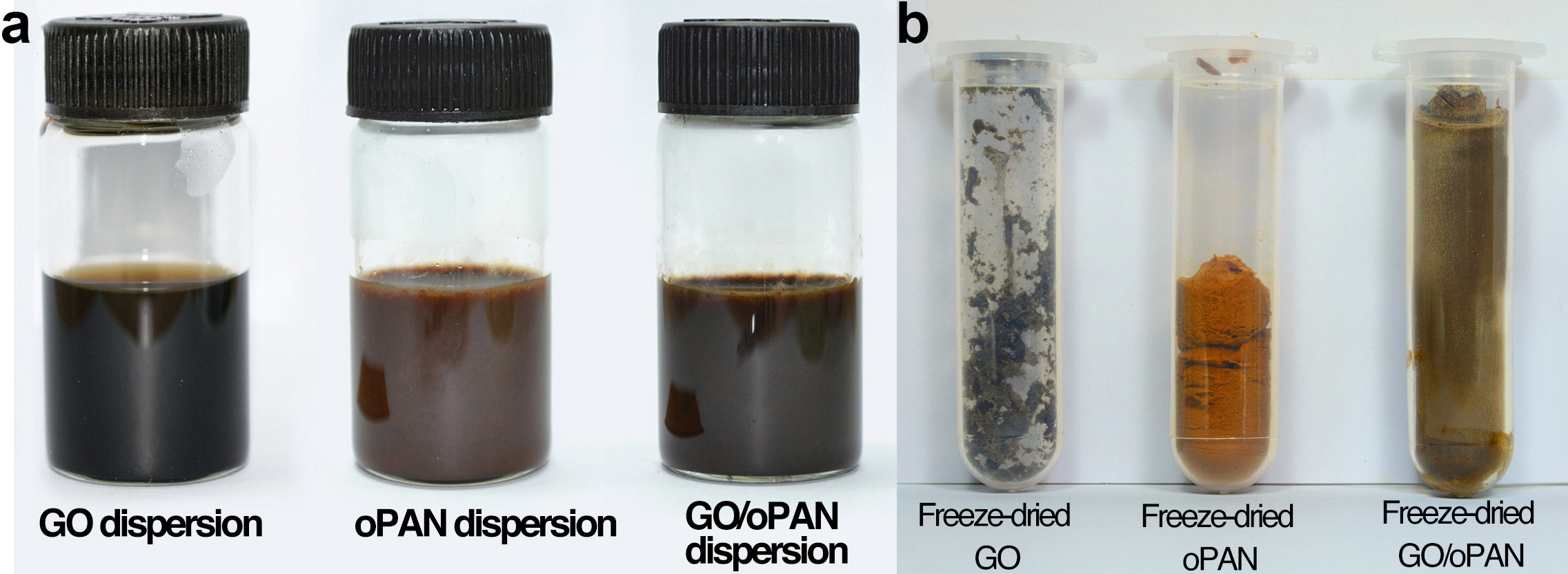


**Figure S4.** (a) Digital photos of GO suspension, oPAN dispersion, GO/oPAN dispersion, and (b) their corresponding products after liquid nitrogen (LN) freezing and freeze-drying.


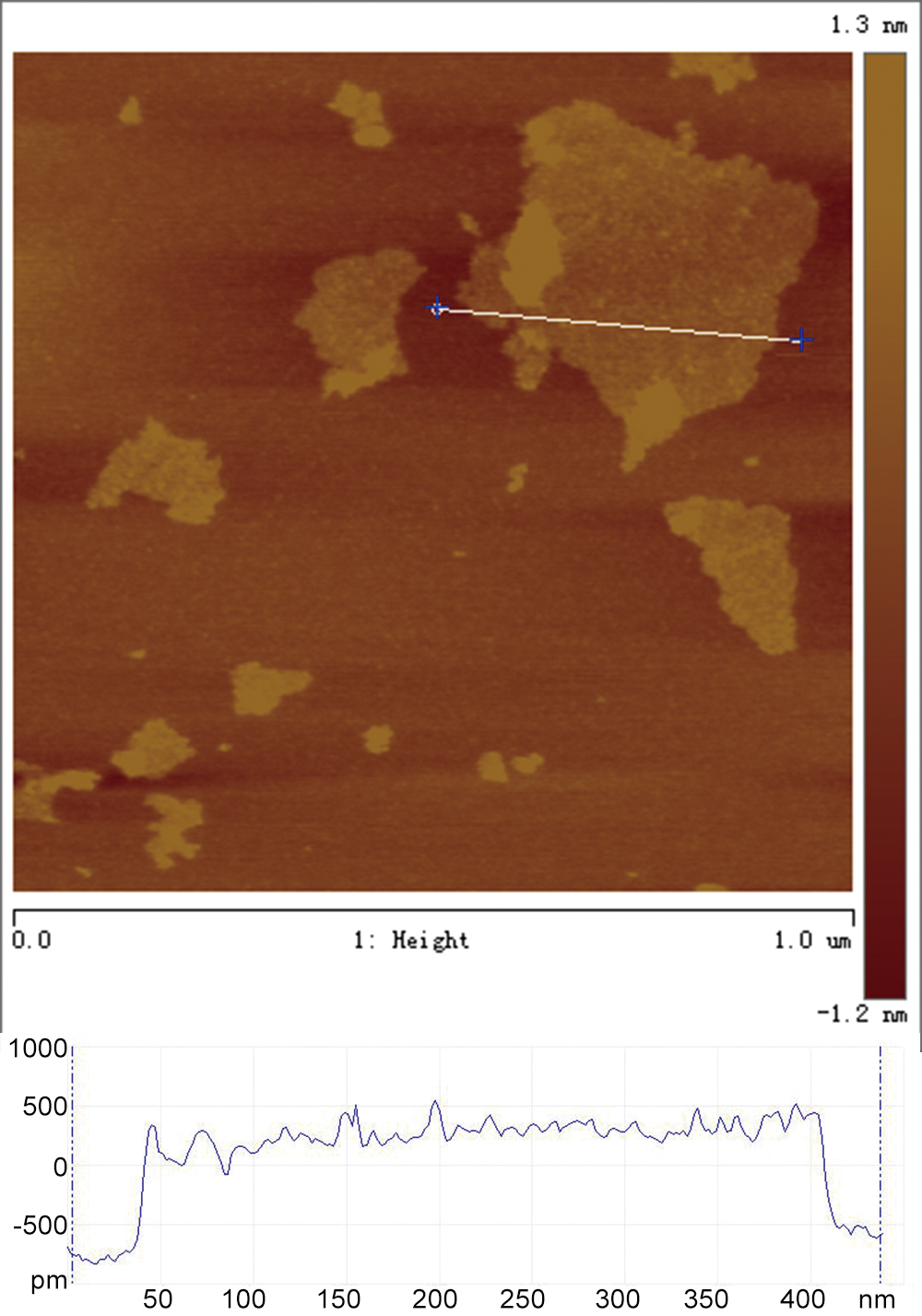


**Figure S5.** AFM image of GO and the corresponding height distribution histogram showing the monolayer graphene oxide with the size range from 50~500 nm.


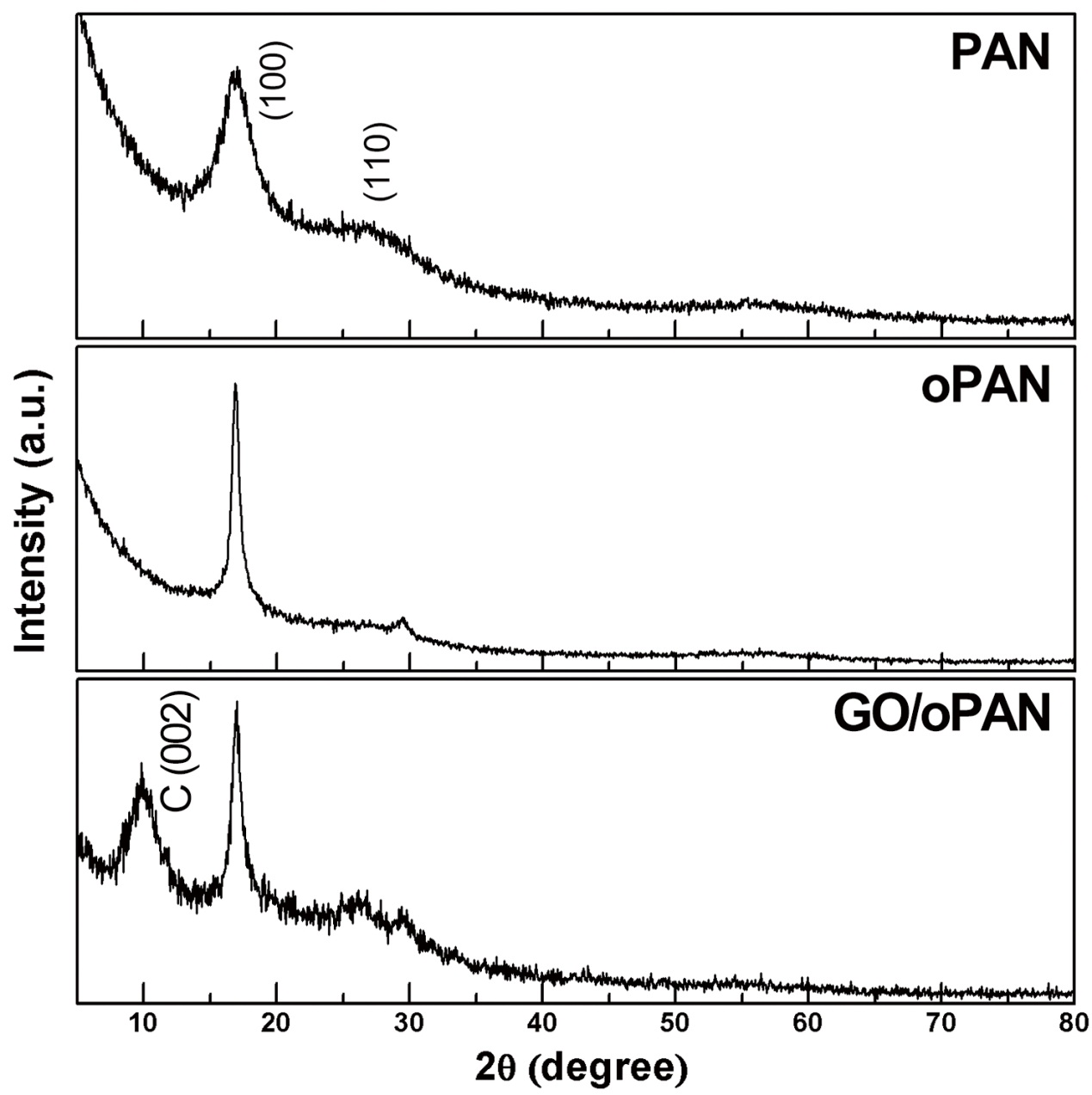


**Figure S6.** XRD patterns of PAN, oPAN membrane and GO/oPAN composites.


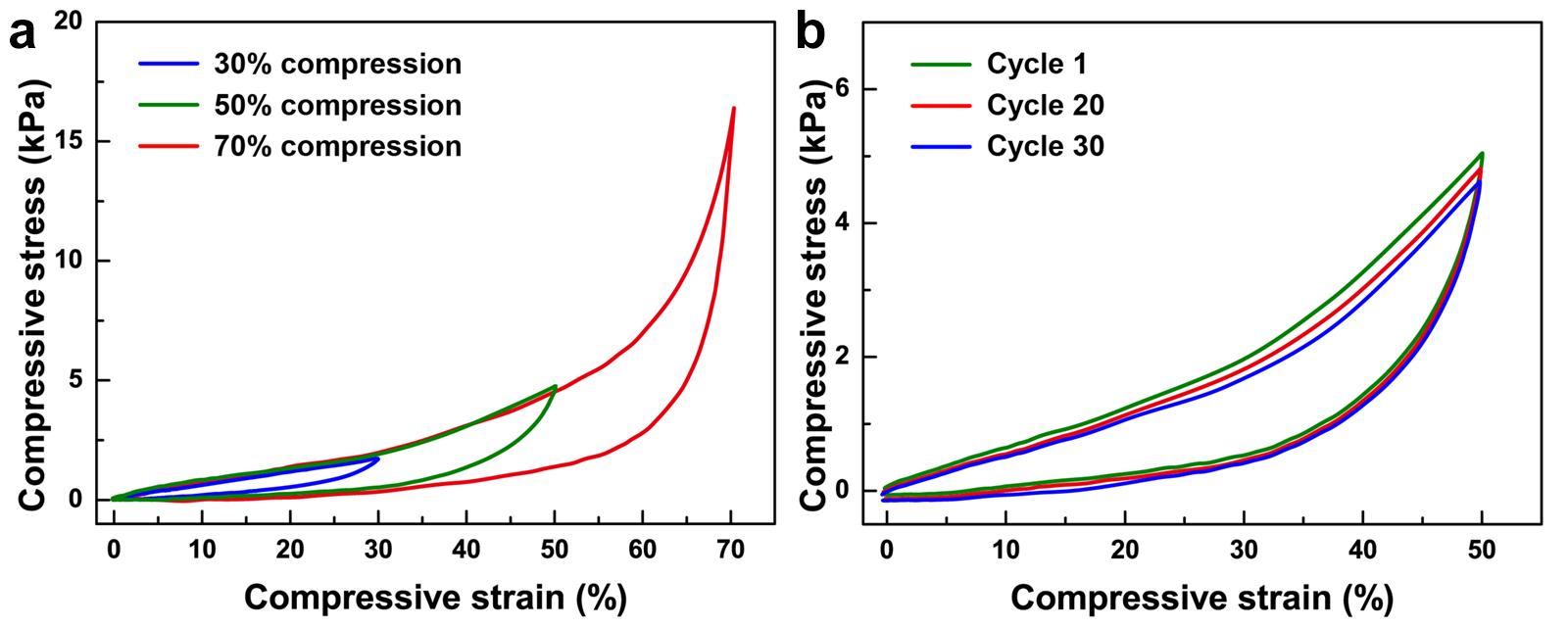


**Figure S7.** (a) Compressive stress–strain curves of GCA at different set strains of 30%, 50%, and 70%. (b) Cyclic stress–strain curves of GCA at a maximum strain of 50% for 30 cycles.


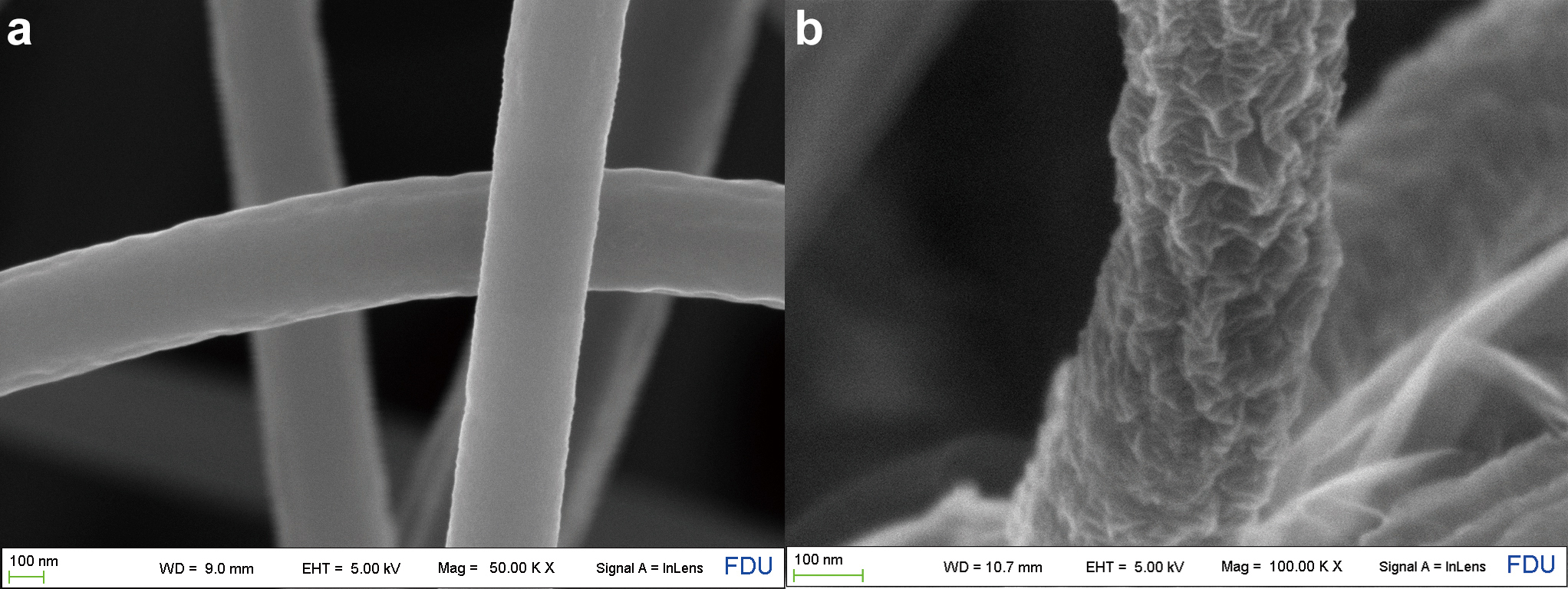


**Figure S8.** SEM images of neat CNFs (a) and graphene wrapped CNFs (b).


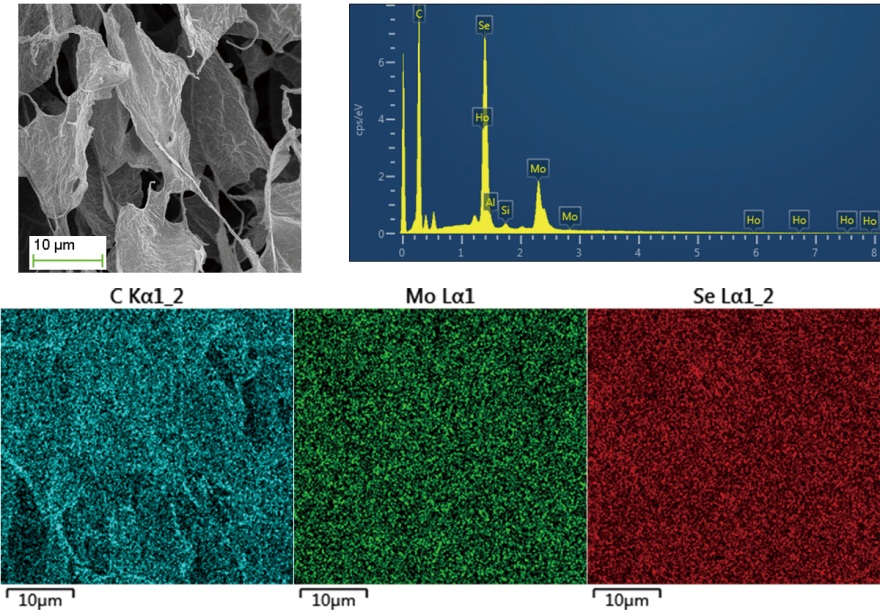


**Figure S9.** EDS elemental mapping of GCAM hybrid.


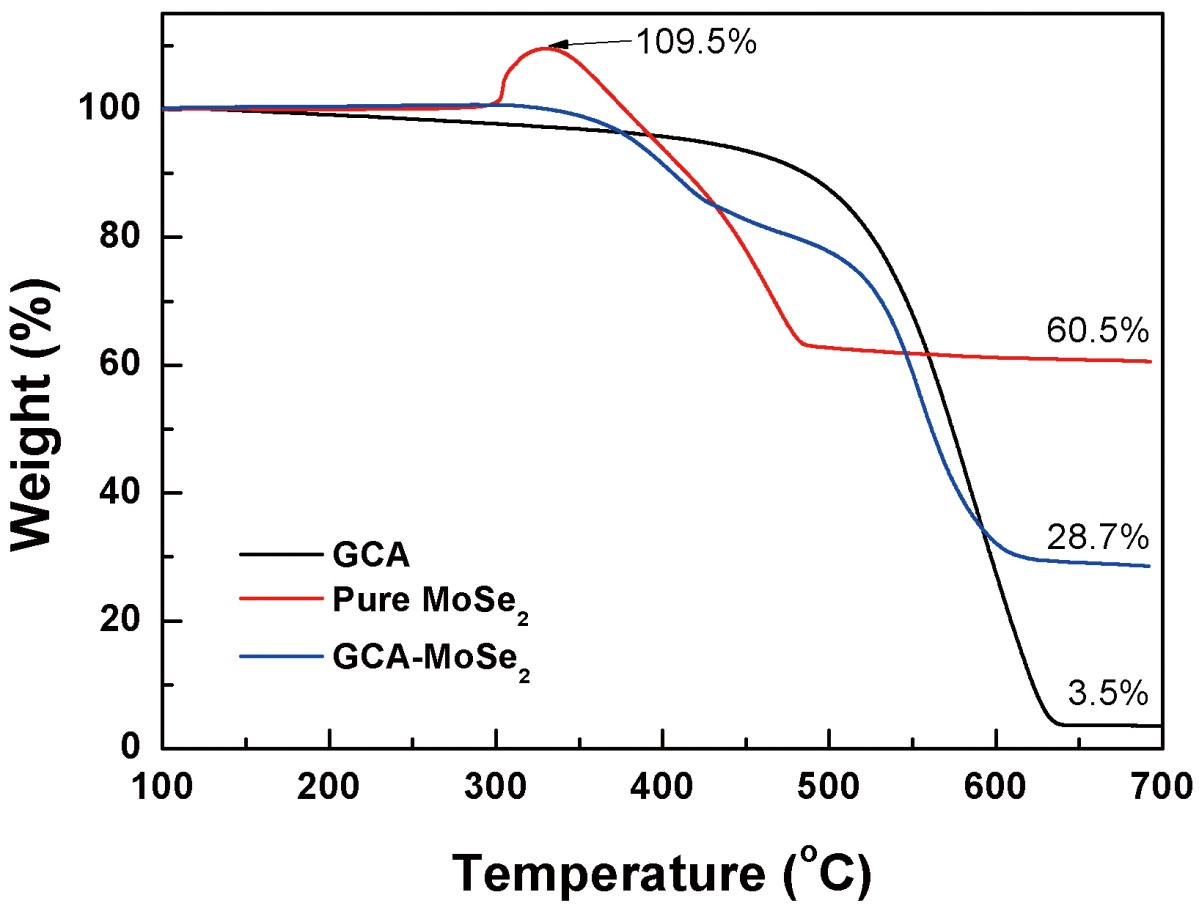


**Figure S10.** TGA curves of GCA, pure MoSe_2_ and GCAM hybrid.


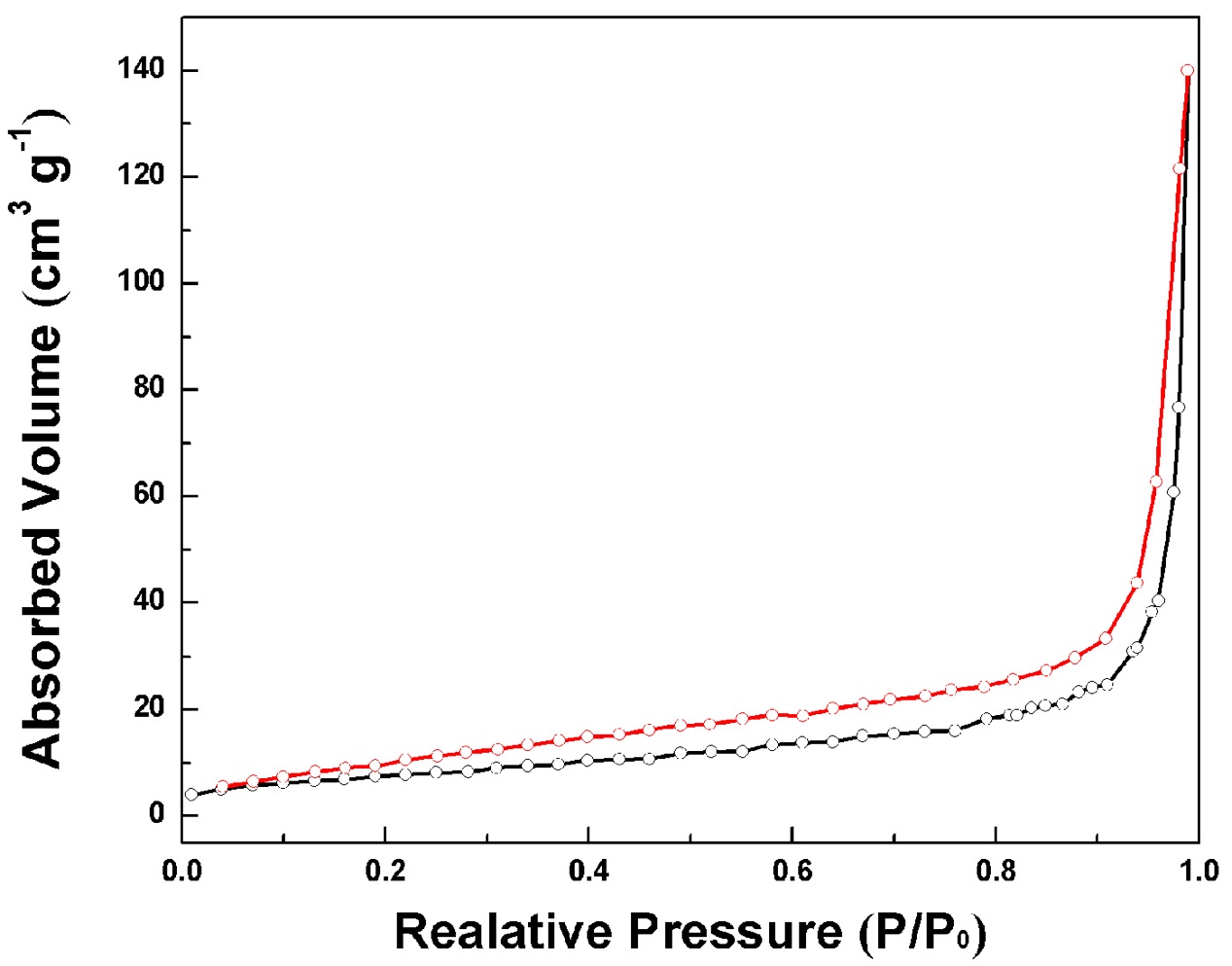


**Figure S11.** Nitrogen adsorption/desorption isotherms of GCA.


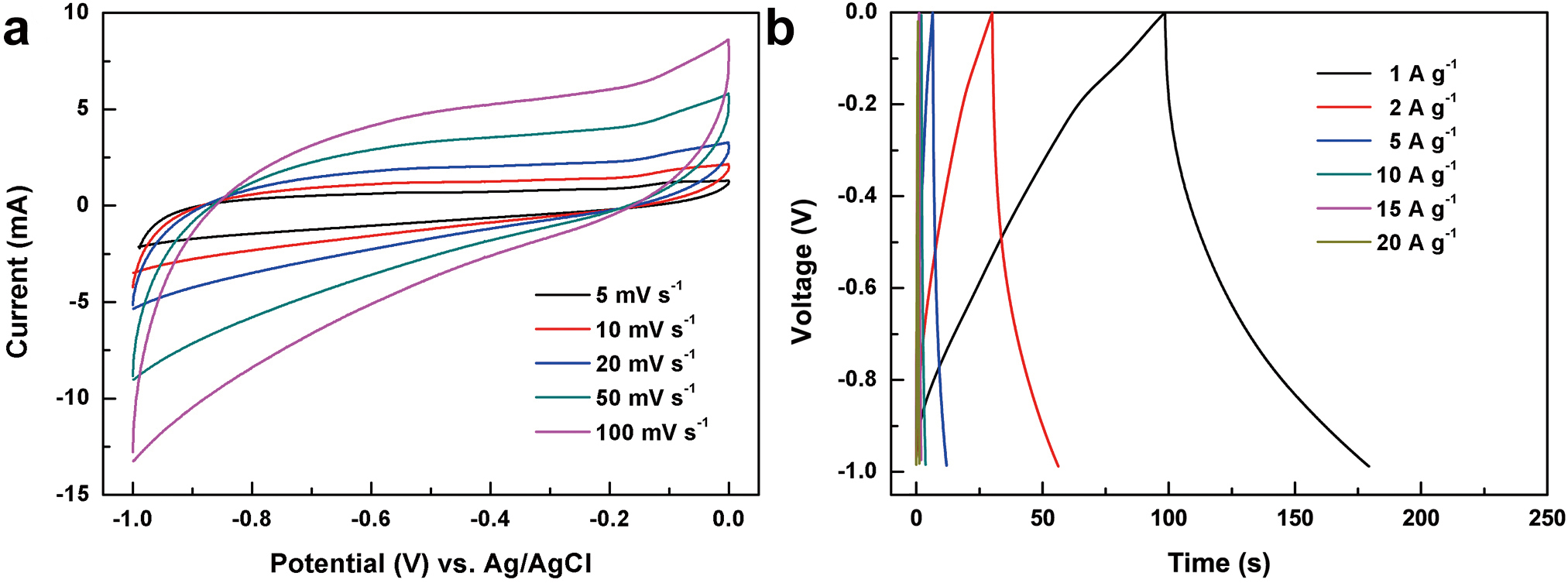


**Figure S12.** CV curves (a) and galvanostatic charge/discharge curves (b) of short CNFs at different scan rates and current densities.


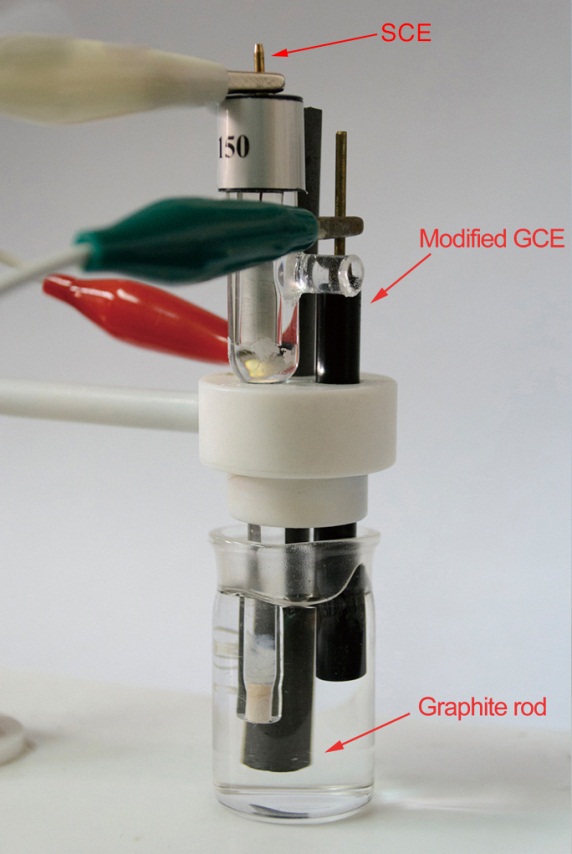


**Figure S13.** Setup for HER tests of GCAM hybrid in both acidic and alkaline solutions.


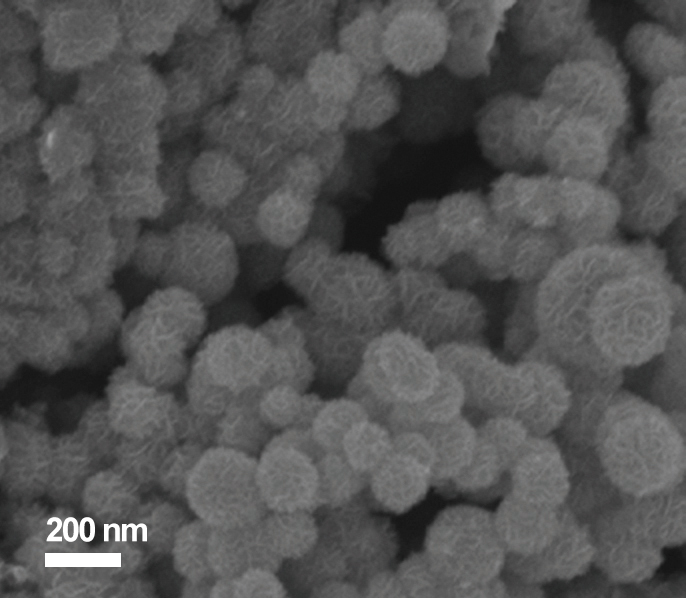


**Figure S14.** SEM image of pure MoSe_2_ spheres synthesized via the same solvothermal method without GCA template.


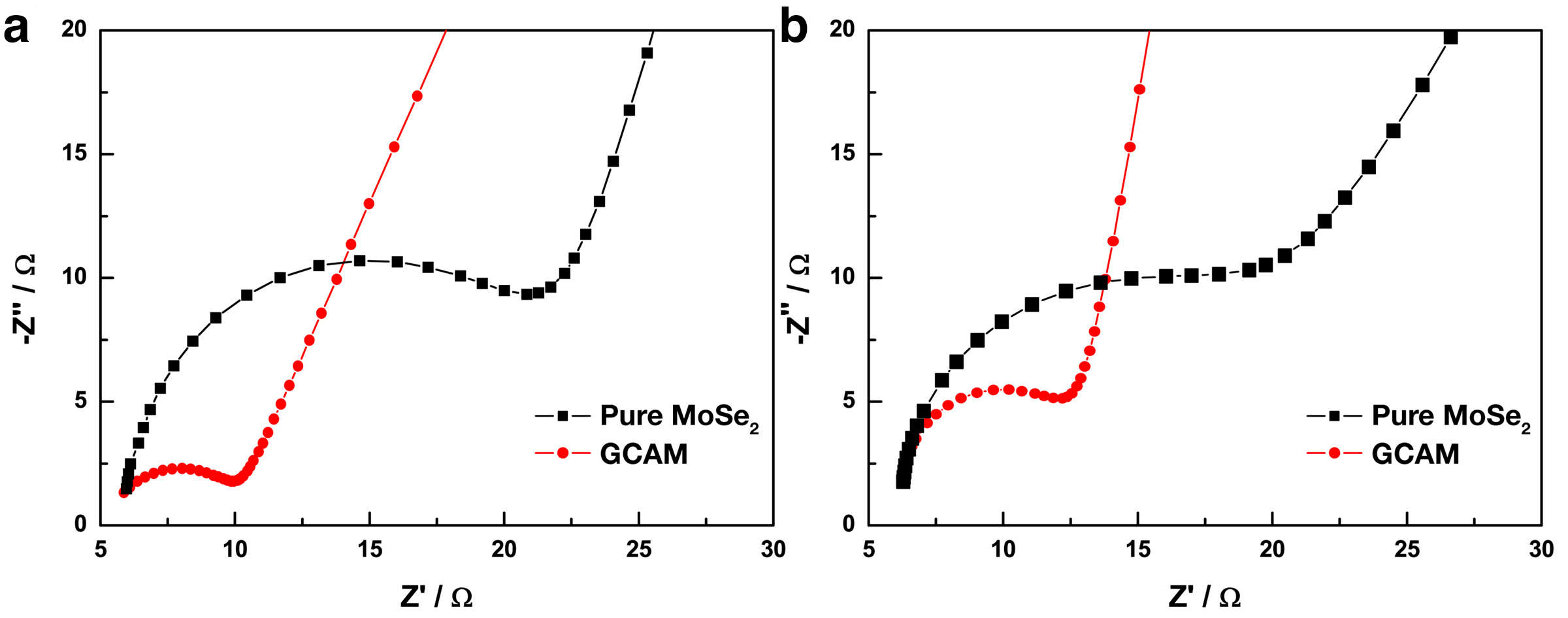


**Figure S15.** Electrochemical impedance spectra of pure MoSe_2_ spheres and GCAM composites in 0.5 M H_2_SO_4_ (a) and 1 M KOH (b).


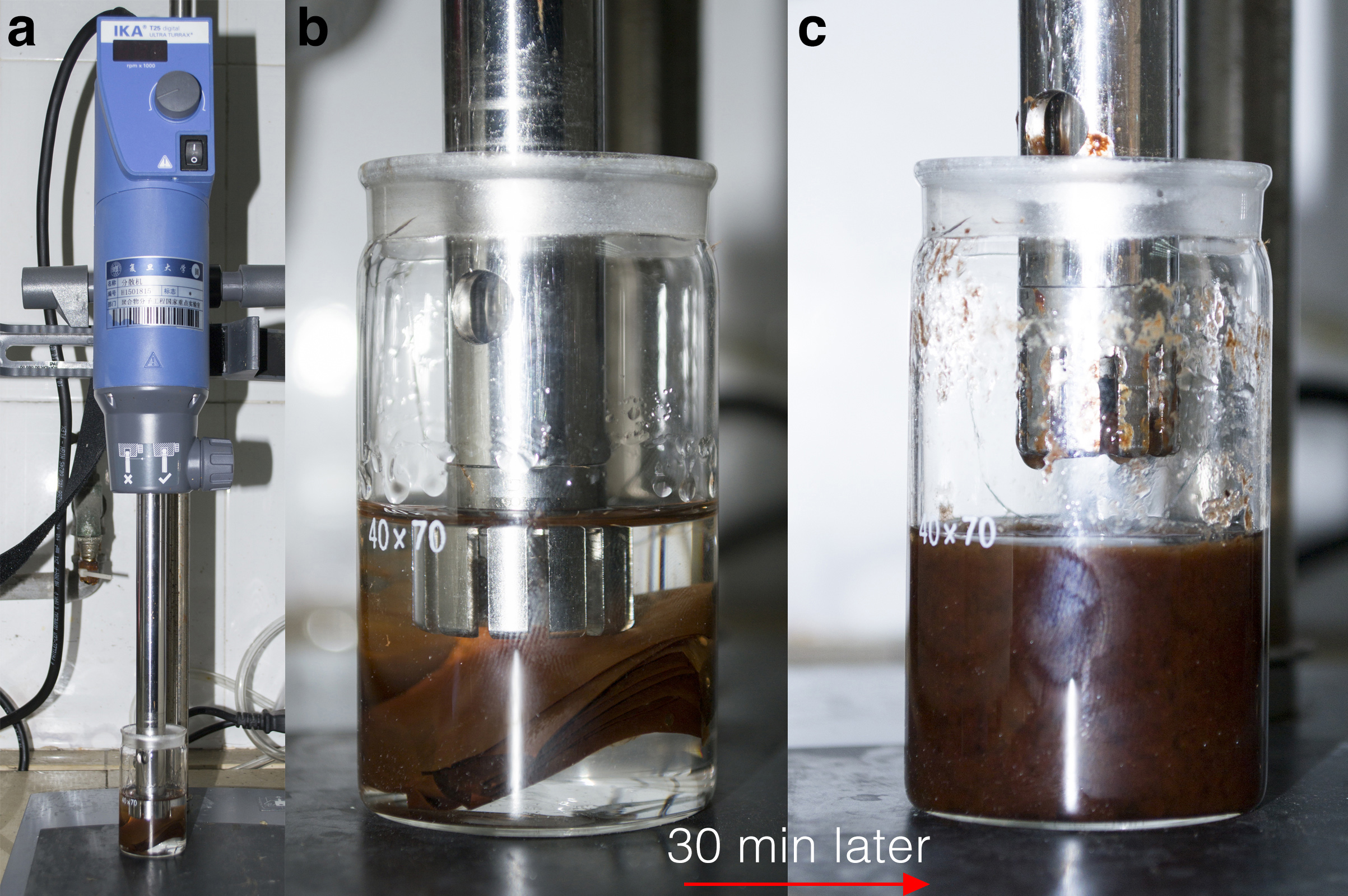


**Figure S16.** Photographs showing the fabrication of oPAN dispersions by high-speed homogenization treatment: (a) IKA T25 homogenizer. (b) oPAN membranes before homogenization treatment. (c) oPAN dispersions after homogenization at 13000 r.p.m for 30 min.
